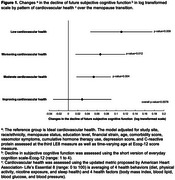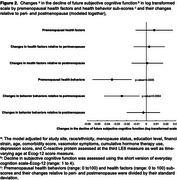# Cardiovascular health over the menopause transition and declines in subjective cognitive function later in life: The Study of Women's Health Across the Nation (SWAN)

**DOI:** 10.1002/alz70860_103069

**Published:** 2025-12-23

**Authors:** Meiyuzhen Qi, Ziyuan Wang, Carol A. Derby, Rebecca C. Thurston, Imke Janssen, Arun S. Karlamangla, Maria M. Brooks, Samar R. El Khoudary

**Affiliations:** ^1^ University of Pittsburgh, Pittsburgh, PA, USA; ^2^ Department of Neurology, and Department of Epidemiology and Population Health, Albert Einstein College of Medicine, Bronx, NY, USA; ^3^ University of Pittsburgh School of Medicine, Pittsburgh, PA, USA; ^4^ Rush University, Chicago, IL, USA; ^5^ University of California Los Angeles, Los Angeles, CA, USA

## Abstract

**Background:**

Over the menopause transition (MT), many women experience accelerated deterioration in their cardiovascular health (CVH), a critical modifiable risk factor for Alzheimer's disease and related dementia (ADRD). Yet, limited research characterized the link between CVH status over the MT and future subjective cognitive function shown to predict and identify ADRD. We evaluated the associations of 1) changes in the Life's Essential 8 (LE8), an established CVH metric, over the MT and 2) LE8 health behavior and health factor sub‐scores with future declines in subjective cognitive function measured by Ecog‐12 (higher score indicates greater declines in function).

**Methods:**

SWAN participants without a history of stroke who had data on LE8 (assessed over 3 visits correspoding to pre‐, peri‐, and postmenopause stages) and Ecog‐12 (measured 1 to 2 times later in the study) were included. CVH status at each visit was classfied as low (<50), moderate (50‐79), or ideal (80) based on the total LE8 score. Changes in CVH over the MT were defined based on pattern of CVH status over time into one of 5 classifications: low remains low (Low), moderate remains moderate (Moderate), ideal remains ideal (Ideal), Improving, or Worsening CVH. Multivariable linear mixed models were used to assess the associations of 1) pattern of CVH status over the MT and 2) premenopausal health behavior and health factor sub‐scores (first LE8) and their changes relative to perimenopause (second LE8) and postmenopause (third LE8) with longitudinal Ecog‐12 scores.

**Results:**

We evaluated 1140 women aged 55.5±2.6, and 65.7±2.7 and 72.2±2.7 years at the third LE8 (analysis baseline), and first and second Ecog‐12 measures, respectively. Compared with women with Ideal CVH, women with Low, Worsening, and Moderate CVH status over the MT had 11%, 7%, and 5% greater declines in subjective cognitive function (log transfromed scale), respectively (Figure 1). Better premenopausal health behavior sub‐scores and greater improvements in health behaviors from pre‐ to perimenopause were associated with less later declines in subjective cognition (Figure 2).

**Conclusions:**

Maintaining good CVH over the MT and adopting healthy behaviors in the transition from pre‐ to perimenopause were associated with less declines in cognitive function later in life.